# Splitting Strategy for Simulating Genetic Regulatory Networks

**DOI:** 10.1155/2014/683235

**Published:** 2014-02-02

**Authors:** Xiong You, Xueping Liu, Ibrahim Hussein Musa

**Affiliations:** Department of Applied Mathematics, Nanjing Agricultural University, Nanjing 210095, China

## Abstract

The splitting approach is developed for the numerical simulation of genetic regulatory networks with a stable steady-state structure. The numerical results of the simulation of a one-gene network, a two-gene network, and a p53-mdm2 network show that the new splitting methods constructed in this paper are remarkably more effective and more suitable for long-term computation with large steps than the traditional general-purpose Runge-Kutta methods. The new methods have no restriction on the choice of stepsize due to their infinitely large stability regions.

## 1. Introduction

The exploration of mechanisms of gene expression and regulation has become one of the central themes in medicine and biological sciences such as cell biology, molecular biology, and systems biology [1, 2]. For example, it has been acknowledged that the p53 tumor suppressor plays key regulatory roles in various fundamental biological processes, including development, ageing, and cell differentiation. It can regulate its downstream genes through their signal pathways and further implement cell cycle arrest and cell apoptosis [3–6]. The qualitative analysis as well as numerical simulation has become an important route in the investigation of differential equations of genetic regulatory networks (GRNs) in the past few years [7–10]. Up till now, algorithms used in the simulation of GRNs have primarily been classical Runge-Kutta (RK) methods (typically of order four) or Runge-Kutta-Fehlberg embedded pairs as employed in the scientific computing software MATLAB [11–13]. However, if we are required to achieve a very high accuracy, we have to take very small stepsize. Moreover, the traditional Runge-Kutta type methods often fail to retain some important qualitative properties of the system of interest. This prevents us from acquiring correct knowledge of the dynamics of genetic regulatory networks.

Geometric numerical integration aims at solving differential equations effectively while preserving the geometric properties of the exact flow [14]. Recently, You et al. [15] develop a family of trigonometrically fitted Scheifele two-step (TFSTS) methods, derive a set of necessary and sufficient conditions for TFSTS methods to be of up to order five based on the linear operator theory, and construct two practical methods of algebraic four and five, respectively. Very recently, You [16] develops a new family of phase-fitted and amplification methods of Runge-Kutta type which have been proved very effective for genetic regulatory networks with a limit-cycle structure.

Splitting is one of the effective techniques in geometric integration. For example, Blanes and Moan [17] construct a symmetric fourth- and sixth-order symplectic partitioned Runge-Kutta and Runge-Kutta-Nyström methods and show that these methods have an optimized efficiency. For a systematic presentation of the splitting technique, the reader is referred to Hairer et al. [14]. The purpose of this paper is to develop the splitting methods for GRNs. In Section 2 we present the system of differential equations governing the GRNs and basic assumptions for the system. In Section 3 we describe the idea and formation of the approach of splitting strategy which intends to simulate exactly the characteristic part of the system.  Section 4 gives the simulation results of the new splitting methods and the traditional Runge-Kutta methods when they are applied to a one-gene network, a two-gene network, and a p53-mdm2 network. We compare their accuracy and computational efficiency.  Section 5 is devoted to conclusive remarks.  Section 6 is for discussions. In Appendix, the linear stability of the new splitting methods is analyzed.

## 2. Materials

### 2.1. mRNA-Protein Networks

An *N*-gene regulatory network can be described by the following system of ordinary differential equations:

(1) $$\begin{array}{c} \dot{r}\left(t\right)=-\Gamma r\left(t\right)+F\left(p\left(t\right)\right),\\ \dot{p}\left(t\right)=-Mp\left(t\right)+Kr\left(t\right), \end{array}$$

where *r*(*t*) = (*r*_1_(*t*),…, *r*_*N*_(*t*)) and *p*(*t*) = (*p*_1_(*t*), *p*_2_(*t*),…, *p*_*N*_(*t*)) are *N*-dimensional vectors representing the concentrations of mRNAs and proteins at time *t*, respectively, and *F*(*p*(*t*)) = (*F*_1_(*p*(*t*)),…, *F*_*N*_(*p*(*t*))), Γ = diag⁡(*γ*_1_,…, *γ*_*N*_), *M* = diag⁡(*μ*_1_,…, *μ*_*N*_), and *K* = diag⁡(*κ*_1_,…, *κ*_*N*_) are diagonal matrices. The system (1) can be written in components as

(2) $$\begin{array}{c} {\dot{r}}_{i}\left(t\right)=-\gamma_{i}r_{i}\left(t\right)+F_{i}\left(p\left(t\right)\right),\\ {\dot{p}}_{i}\left(t\right)=-\mu_{i}p_{i}\left(t\right)+\kappa_{i}r_{i}\left(t\right), \end{array}$$

where, for *i* = 1,2,…, *N*, *r*_*i*_(*t*) and *p*_*i*_(*t*) ∈ ℝ^+^ are the concentrations of mRNA *i* and the corresponding protein *i* at time *t*, respectively; *γ*_*i*_ and *μ*_*i*_, positive constants, are the degradation rates of mRNA *i* and protein *i*, respectively; *κ*_*i*_ is a positive constant; and *F*_*i*_(·), the regulatory function of gene *i*, is a nonlinear and monotonic function in each of its variables. If gene *i* is activated by protein *j*, ∂*F*_*i*_/∂*p*_*j*_ > 0, and if gene *i* is inhibited by protein *j*, ∂*F*_*i*_/∂*p*_*j*_ < 0. If protein *j* has no action on gene *i*, *p*_*j*_ will not appear in the expression of *F*_*i*_.

In particular, we are concerned in this paper with the following two simple models.

(I) The first model is a one-gene regulatory network which can be written as

(3) $$\begin{array}{c} \dot{r}\left(t\right)=-\gamma r\left(t\right)+f\left(p\left(t\right)\right),\\ \dot{p}\left(t\right)=-\mu p\left(t\right)+\kappa r\left(t\right), \end{array}$$

where *f*(*p*(*t*)) = *α*/(1 + *p*(*t*)^2^/*θ* ^2^) represents the action of an inhibitory protein that acts as a dimer and *γ*, *μ*, *κ*, *α*, and *θ* are positive constants. This model with delays can be found in Xiao and Cao [18].

(II) The second model is a two-gene cross-regulatory network [7, 19]:

(4) $$\begin{array}{c} {\dot{r}}_{1}=\lambda_{1}h^{+}\left(p_{2};\theta_{2},n_{2}\right)-\gamma_{1}r_{1},\\ {\dot{r}}_{2}=\lambda_{2}h^{-}\left(p_{1};\theta_{1},n_{1}\right)-\gamma_{2}r_{2},\\ {\dot{p}}_{1}=\kappa_{1}r_{1}-\delta_{1}p_{1},\\ {\dot{p}}_{2}=\kappa_{2}r_{2}-\delta_{2}p_{2}, \end{array}$$

where *r*_1_ and *r*_2_ are the concentrations of mRNA 1 and mRNA 2, respectively, *p*_1_ and *p*_2_ are the concentrations of their corresponding products protein 1 and protein 2, respectively, *λ*_1_, and *λ*_2_ represent the maximal transcription rates of gene 1 and gene 2, respectively, *γ*_1_ and *γ*_2_ are the degradation rates of mRNA 1 and mRNA 2, respectively, *δ*_1_ and *δ*_2_ are the degradation rates of protein 1 and protein 2, respectively,

(5) $$\begin{array}{c} h^{+}\left(p_{2};\theta_{2},n_{2}\right)=\frac{p_{2}^{n_{2}}}{p_{2}^{n_{2}}+\theta_{2}^{n_{2}}},\\ h^{-}\left(p_{1};\theta_{1},n_{1}\right)=\frac{\theta_{1}^{n_{1}}}{p_{1}^{n_{1}}+\theta_{1}^{n_{1}}} \end{array}$$

are the Hill functions for activation and repression, respectively, *n*_1_ and *n*_2_ are the Hill coefficients, and *θ*_1_ and *θ*_2_ are the thresholds. It is easy to see that the activation function *h* ^+^ is increasing in *p*_2_ and the repression function *h* ^−^ is decreasing in *p*_1_.

### 2.2. A p53-mdm2 Regulatory Pathway

Another model we are interested in is for the damped oscillation of the p53-mdm2 regulatory pathway which is given by (see [20])

(6) $$\begin{array}{c} {\dot{P}}_{I}=s_{p}+j_{a}P_{A}-\left(d_{p}+k_{a}S\left(t\right)\right)P_{I}-k_{c}P_{I}M+j_{c}C,\\ \dot{M}=s_{m0}+\frac{s_{m1}P_{I}+s_{m2}P_{A}}{P_{I}+P_{A}+K_{m}}+k_{u}C+j_{c}C-\left(d_{m}+k_{c}P_{I}\right)M,\\ \dot{C}=k_{c}P_{I}M-\left(j_{c}+k_{u}\right)C,\\ {\dot{P}}_{A}=k_{a}S\left(t\right)P_{I}-\left(j_{a}+d_{p}\right)P_{A}, \end{array}$$

where *P*_*I*_ represents the concentration of the p53 tumour suppressor, *M* (mdm2) is the concentration of the p53's main negative regulator, *C* is the concentration of the p53-mdm2 complex, *P*_*A*_ is the concentration of an active form of p53 that is resistant against mdm2-mediated degradation, *S*(*t*) is a transient stress stimulus which has the form *S*(*t*) = −*e* ^*c*_*s*_*t*^,  *c*_*s*_ = *γk*_*u*_, *s*_∗_(∗ = *p*, *m*0, *m*1) are *de novo* synthesis rates, *k*_∗_(∗ = *a*, *c*, *u*) are production rates, *j*_∗_(∗ = *a*, *c*) are reverse reactions (e.g., dephosphorylation), *d*_*p*_ is the degradation rate of active p53, and *K*_*m*_ is the saturation coefficient.

## 3. Methods

### 3.1. Runge-Kutta Methods

Either the mRNA-protein network (1) or the p53-mdm2 regulatory pathway (6) can be regarded as a special form of a system of ordinary differential equations (ODEs):

(7) $$\begin{array}{c} z^{\prime }=g\left(z\right), \end{array}$$

where *z* = *z*(*t*) ∈ *R* ^*d*^ and the function *g* : ℝ^*d*^ → ℝ^*d*^ is smooth enough as required. The system (7) together with initial value *z*(0) = *z*_0_ is called an *initial value problem* (IVP). Throughout this paper we make the following assumptions.

- The system (7) has a unique positive steady state z*; that is, there is a unique z* = (*z*_1_*, *z*_2_*,…, *z*_d_*) with *z*_i_* > 0 for *i* = 1,2,…, *d* such that *g*(z*) = 0.
- The steady state z* is asymptotically stable; that is, for any solution *z*(*t*) of the system (7) through a positive initial point *z*_0_, lim⁡_*t*→+*∞*_⁡*z*(*t*) = z*.

The most frequently used algorithms for the system (7) are the so-called Runge-Kutta methods which read

(8) $$\begin{array}{c} Z_{i}=z_{n}+h\overset{s}{\underset{j=1}{\sum }}a_{ij}g\left(Z_{j}\right),\;i=1,\ldots ,s,\\ z_{n+1}=z_{n}+h\overset{s}{\underset{i=1}{\sum }}b_{i}g\left(Z_{i}\right), \end{array}$$

where *z*_*n*_ is an approximation of the solution *z*(*t*) at *t*_*n*_,  *n* = 0,1,…, *a*_*ij*_, *b*_*i*_,  *i*, *j* = 1,…, *s*, are real numbers, *s* is the number of internal stages *Z*_*i*_, and *h* is the step size. The scheme (8) can be represented by the Butcher tableau:

(9) $$\begin{array}{c} \begin{array}{ccc} \begin{array}{cc} c & A\\ & b^{T} \end{array} & =\begin{array}{cccc} c_{1} & a_{11} & \ldots & a_{1s}\\ \vdots & \vdots & \ddots & \vdots \\ c_{s} & a_{s1} & \ldots & a_{ss}\\ & b_{1} & \ldots & b_{s} \end{array} & \end{array} \end{array}$$

or simply by (*c*, *A*, *b*(*ν*)), where *c*_*i*_ = ∑_*j*=1_^*s*^ *a*_*ij*_ for *i* = 1,…, *s*. Two of the most famous fourth-order RK methods have the tableaux as follows (see [13]):


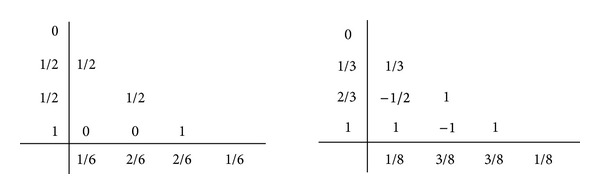
(10)

which we denote as RK4 and RK3/8, respectively.

### 3.2. Splitting Methods

Splitting methods have been proved to be an effective approach to solve ODEs. The main idea is to split the vector field into two or more integrable parts and treat them separately. For a concise account of splitting methods, see Chapter II of Hairer et al. [14].

Suppose that the vector field *g* of the system (7) has a split structure

(11) $$\begin{array}{c} z^{\prime }=g^{\left[1\right]}\left(z\right)+g^{\left[2\right]}\left(z\right). \end{array}$$

Assume also that both systems *z*′ = *g* ^[1]^(*z*) and *z*′ = *g* ^[2]^(*z*) can be solved in closed form or are accurately integrated and their exact flows are denoted by *φ*_*h*_^[1]^ and *φ*_*h*_^[2]^, respectively.

**Definition 1**

(i) The method defined by

(12) $$\begin{array}{c} \Psi_{h}=\varphi_{h}^{\left[2\right]}\circ \varphi_{h}^{\left[1\right]},\\ \Phi_{h}=\varphi_{h}^{\left[1\right]}\circ \varphi_{h}^{\left[2\right]} \end{array}$$

is the simplest splitting method for the system (7) based on the decomposition (11) (see [13]).

(ii) The Strang splitting is the following symmetric version (see [21]):

(13) $$\begin{array}{c} \Psi_{h}=\varphi_{h/2}^{\left[1\right]}\circ \varphi_{h}^{\left[2\right]}\circ \varphi_{h/2}^{\left[1\right]}. \end{array}$$

(iii) The general splitting method has the form

(14) $$\begin{array}{c} \Psi_{h}=\varphi_{b_{m}h}^{\left[2\right]}\circ \varphi_{a_{m}h}^{\left[1\right]}\circ \cdots \circ \varphi_{b_{2}h}^{\left[2\right]}\circ \varphi_{a_{2}h}^{\left[1\right]}\circ \varphi_{b_{1}h}^{\left[2\right]}\circ \varphi_{a_{1}h}^{\left[1\right]}, \end{array}$$

where *a*_1_, *b*_1_, *a*_2_, *b*_2_,…, *a*_*m*_, *b*_*m*_ are positive constants satisfying some appropriate conditions. See, for example, [22–25].

Theorem 5.6 in Chapter II of Hairer et al. [14] gives the conditions for the splitting method (14) to be of order *p*.

However, in most occasions, the exact flows *φ*_*h*_^[1]^ and *φ*_*h*_^[2]^ for *g* ^[1]^ and *g* ^[2]^ in Definition 1 are not available. Hence, we have to use instead some approximations or numerical flows which are denoted by *ψ*_1_ and *ψ*_2_.

### 3.3. Splitting Methods for Genetic Regulatory Networks Based on Their Characteristic Structure

For a given genetic regulatory network, different ways of decomposition of the vector field *f* may produce different results of computation. Thus a question arises as follows: which decomposition is more appropriate or more effective. In the following we take the system (1) for example. The analysis of the p53-mdm2 pathway (6) is similar. Denote *z*(*t*) = (*r*(*t*), *p*(*t*))^*T*^. Then the *N*-gene regulatory network (1) has a natural form of decomposition:

(15) $$\begin{array}{c} g^{\left[1\right]}\left(z\right)=\left(\begin{array}{cc} -\Gamma & 0\\ K & -M \end{array}\right)z,\;\;g^{\left[2\right]}\left(z\right)=\left(\begin{array}{c} F\left(p\left(t\right)\right)\\ 0 \end{array}\right). \end{array}$$

Unfortunately, it has been checked through practical test that the splitting methods based on this decomposition cannot lead to effective results. To find a way out, we first observe that a coordinate transform *x*(*t*) = *r*(*t*) − r*, *y*(*t*) = *p*(*t*) − p* translates the steady state (r*, p*) of the system (1) to the origin and yields

(16) $$\begin{array}{c} \dot{x}\left(t\right)=-\Gamma x\left(t\right)+F^{\prime }\left(p^{*}\right)y\left(t\right)+G\left(y\left(t\right)\right),\\ \dot{y}\left(t\right)=Kx\left(t\right)-My\left(t\right), \end{array}$$

where *F*′(p*) is the Jacobian matrix of *F*(*p*) at point p* and *G*(*y*(*t*)) = *F*(p* + *y*(*t*)) − *F*′(p*)*y*(*t*)  −  *F*(p*). We employ this special structure of the system (16) to reach the decomposition of the vector field:

(17) $$\begin{array}{c} g^{\left[1\right]}\left(z\right)=\left(\begin{array}{cc} -\Gamma & F^{\prime }\left(p^{*}\right)\\ K & -M \end{array}\right)z,\;\;g^{\left[2\right]}\left(z\right)=\left(\begin{array}{c} G\left(y\left(t\right)\right)\\ 0 \end{array}\right), \end{array}$$

where *z*(*t*) = (*x*(*t*), *y*(*t*))^*T*^. The system $\dot{z}=g^{\left[1\right]}(z)$ here is called the *linearization* of the system (1) at the steady state (r*, p*). *g* ^[1]^ in (17) is the linear principal part of the vector field *g* which has the exact flow $\varphi_{h}^{\left[1\right]}=\exp\left(h\left(\begin{array}{cc} -\Gamma & F^{\prime }\left(p^{*}\right)\\ K & -M \end{array}\right)\right)$. However, it is not easy or impossible to obtain the exact solution of *g* ^[2]^(*x*_*i*_(*t*), *y*_*i*_(*t*)) due to its nonlinearity. So we have to use an approximation flow *ψ*_*h*_^[2]^ and form the splitting method:

(18) $$\begin{array}{c} \Psi_{h}=\psi_{h}^{\left[2\right]}\circ \varphi_{h}^{\left[1\right]}. \end{array}$$

When *ψ* ^[2]^ is taken as an RK method, then the resulting splitting method is denoted by Split(Exact:RK). Hence we write Split(Exact:RK4) and Split(Exact:RK3/8) for the splitting methods with *ψ* ^[2]^ taken as RK4 and RK3/8, respectively.

## 4. Results

In order to examine the numerical behavior of the new splitting methods Split(Exact:RK4) and Split(Exact:RK3/8), we apply them to the three models presented in Section 2. Their corresponding RK methods RK4 and RK3/8 are also used for comparison. We will carry out two observations: effectiveness and efficiency. For effectiveness, we first find the errors produced by each method with different values of stepsize. We also solve each problem with a fixed stepsize on different lengths of time intervals.

### 4.1. The One-Gene Network

Table 1 gives the parameter values which are provided by Xiao and Cao [18]. This system has a unique steady state (r*, p*) = (0.6,2) where the Jacobian matrix has eigenvalues *ω*_1_ = −1.2500 + 2.9767*i*,  *ω*_2_ = −1.2500 − 2.9767*i*, where *i* is the imaginary unit satisfying *i* ^2^ = −1. Since the two eigenvalues both have negative real parts, the steady state is asymptotically stable.

In order to apply the splitting methods Split(Exact:RK4) and Split(Exact:RK3/8), the vector field of the system (3) is decomposed in the way of (16) as

(19) g[1](z(t))=(−γ−2αp∗/θ2(1+p∗2/θ2)2κ−μ)(x(t)y(t)),g[2](z(t)) =(α1+(p∗+y(t))2/θ2+2αp∗/θ2(1+p∗2/θ2)2y(t)0).

The system is solved on the time interval [0,100] with initial values of mRNA and protein *r*(0) = 0.6,  *p*(0) = 0.8 and with different stepsizes. The numerical results are presented in Table 2.

Then we solve the problem with a fixed stepsize *h* = 2 on several lengths of time intervals. The numerical results are given in Table 3.

### 4.2. The Two-Gene Network

Table 4 gives the parameter values which can be found in Widder et al. [19]. This system has a unique steady state (*r*_1_*, *r*_2_*, *p*_1_*, *p*_2_*) = (0.814713,0.614032,0.814713,0.614032). Since the four eigenvalues *ω*_1_ = −1.9611 + 0.9611*i*,  *ω*_2_ = −1.9611 − 0.9611*i*,  *ω*_3_ = −0.0389 + 0.9611*i*, and *ω*_4_ = −0.0389 − 0.9611*i* of the Jacobian matrix at the steady state (r*, p*) all have negative real parts, the steady state is asymptotically stable.

For this system, the decomposition (16) becomes

(20) g[1](z(t)) =(−γ100n2λ1θ2n2p2∗n2−1(p2∗n2+θ2n2)20−γ2−n1λ2θ1n1p1∗n1−1(p1∗n1+θ1n1)20κ10−δ100κ20−δ2)  ×(x1(t)x2(t)y1(t)y2(t)),g[2](z(t)) =(λ1(y2(t)+p2∗)n2(y2(t)+p2∗)n2+θ2n2−n2λ1θ2n2p2∗n2−1(p2∗n2+θ2n2)2y2(t)λ2θ1n1(y1(t)+p1∗)n1+θ1n1+n1λ2θ1n1p1∗n1−1(p1∗n1+θ1n1)2y1(t)00).

The system is solved on the time interval [0,100] with the initial values *r*_1_(0) = 0.6,  *r*_2_(0) = 0.8,  *p*_1_(0) = 0.6, and *p*_2_(0) = 0.8 and with different stepsizes. The numerical results are presented in Table 5.

Then we solve the problem with a fixed stepsize *h* = 2 on several lengths of time intervals. The numerical results are given in Table 6.

### 4.3. The p53-mdm2 Network

Table 7 gives the parameter values which are used by van Leeuwen et al. [20]. For simplicity, we take the small function *S*(*t*) ≡ 0. This system has a unique steady state (*P*_I_*, M*, C*, *P*_A_*  = (9.42094,0.0372868,3.49529,0). Since the eigenvalues *ω*_1_ = −38.4766,  *ω*_2_ = −0.0028 + 0.0220*i*,  *ω*_3_ = −0.0028 − 0.0220*i*, and *ω*_4_ = −0.2002 of the Jacobian matrix at the steady state all have negative real parts, the steady state is asymptotically stable.

For this system, decomposition (16) becomes

(21) $$\begin{array}{c} g^{\left[1\right]}\left(z\left(t\right)\right)=J\left(\begin{array}{c} z_{1}\left(t\right)\\ z_{2}\left(t\right)\\ z_{3}\left(t\right)\\ z_{4}\left(t\right) \end{array}\right),\\ g^{\left[2\right]}\left(z\left(t\right)\right)=\left(\begin{array}{c} -k_{c}z_{1}\left(t\right)z_{2}\left(t\right)\\ \upsilon \left(t\right)\\ k_{c}z_{1}\left(t\right)z_{2}\left(t\right)\\ 0 \end{array}\right), \end{array}$$

where

(22) J=(−kcM∗−dp−kcPI∗jcjasm1(PA∗+Km)−sm2PA∗(PI∗+PA∗+Km)2−kcM∗−dm−kcPI∗ku+jcsm2(PI∗+Km)−sm1PI∗(PI∗+PA∗+Km)2kcM∗kcPI∗−(jc+ku)0000−(ja+dp)),υ(t)=sm0+sm1(z1(t)+PI∗)+sm2(z4(t)+PA∗)z1(t)+PI∗+z4(t)+PA∗+Km−dmM∗+kcz1(t)z2(t)+2kcz1(t)M∗−kcPI∗M∗−sm1(PA∗+Km)−sm2PA∗(PI∗+PA∗+Km)2z1(t)−sm2(PI∗+Km)−sm1PI∗(PI∗+PA∗+Km)2z4(t)+(ku+jc)C∗.

The system is solved on the time interval [0,100] with initial values *P*_*I*_(0) = 9.42 nM, *C*(0) = 0.037 nM, *M*(0) = 3.49 nM, and *P*_*A*_(0) = 0 nM and with different stepsizes. The numerical results are presented in Table 8.

Then we solve the problem with a fixed stepsize *h* = 2 on several lengths of time intervals. The numerical results are given in Table 9.

## 5. Conclusions

In this paper we have developed a new type of splitting algorithms for the simulation of genetic regulatory networks. The splitting technique has taken into account the special structure of the linearizing decomposition of the vector field. From the results of numerical simulation of Tables 2, 5, and 8, we can see that the new splitting methods Split(Exact:RK4) and Split(Exact:RK3/8) are much more accurate than the traditional Runge-Kutta methods RK4 and RK3/8. For large steps when RK4 and RK3/8 completely lose effect, Split(Exact:RK4) and Split(Exact:RK3/8) continue to work very well. On the other hand, Tables 3, 6, and 9 show that for comparatively large steps, RK4 and RK3/8 can solve the problem only on short time intervals while Split(Exact:RK4) and Split(Exact:RK3/8) work for very long time intervals.

We conclude that, for genetic regulatory networks with an asymptotically stable steady state, compared with the traditional Runge-Kutta, the new splitting methods have two advantages.

- They are extremely accurate for large steps. This promises high efficiency for solving large-scale systems (complex networks containing a large number of distinct proteins) in a long-term simulation.
- They work effectively for very long time intervals. This makes it possible for us to explore the long-run behavior of genetic regulatory network which is important in the research of gene repair and gene therapy.

The special structure of the new splitting methods and their remarkable stability property (see Appendix) are responsible for the previous two advantages.

## 6. Discussions

The splitting methods designated in this paper have opened a novel approach to effective simulation of the complex dynamical behaviors of genetic regulatory network with a characteristic structure. It is still possible to enhance the effectiveness of the new splitting methods. For example, higher-order splitting methods can be obtained by recursive composition (14) or by employing higher order Runge-Kutta methods; see II.5 of [13]. Another possibility is to consider embedded pairs of two splitting methods which can improve the efficiency; see II.4 of [13].

The genetic regulatory networks considered in this paper are nonstiff. For stiff systems (whose Jacobian possesses eigenvalues with large negative real parts or with purely imaginary eigenvalues of large modulus), the previous techniques suggested by the reviewer are applicable. Moreover, the error control technique which can increase the efficiency of the methods is an interesting theme for future work.

There are more qualitative properties of the genetic regulatory networks that can be taken into account in the designation of simulation algorithms. For example, oscillation in protein levels is observed in most regulatory networks. Symmetric and symplectic methods have been shown to have excellent numerical behavior in the long-term integration of oscillatory systems even if they are not Hamiltonian systems. A brief account of symmetric and symplectic extended Runge-Kutta-Nyström (ERKN) methods for oscillatory Hamiltonian systems and two-step ERKN methods can be found, for instance, in Yang et al. [26], Chen et al. [27], Li et al. [28], and You et al. [29].

Finally, a problem related to this work remains open. We observe that, in Tables 3 and 9 for the p53-mdm2 pathway, as the time interval extends, the error produced by Split(Exact:RK4) and Split(Exact:RK3/8) becomes even smaller. This phenomenon is yet to be explained.

## Figures and Tables

**Figure 1:**
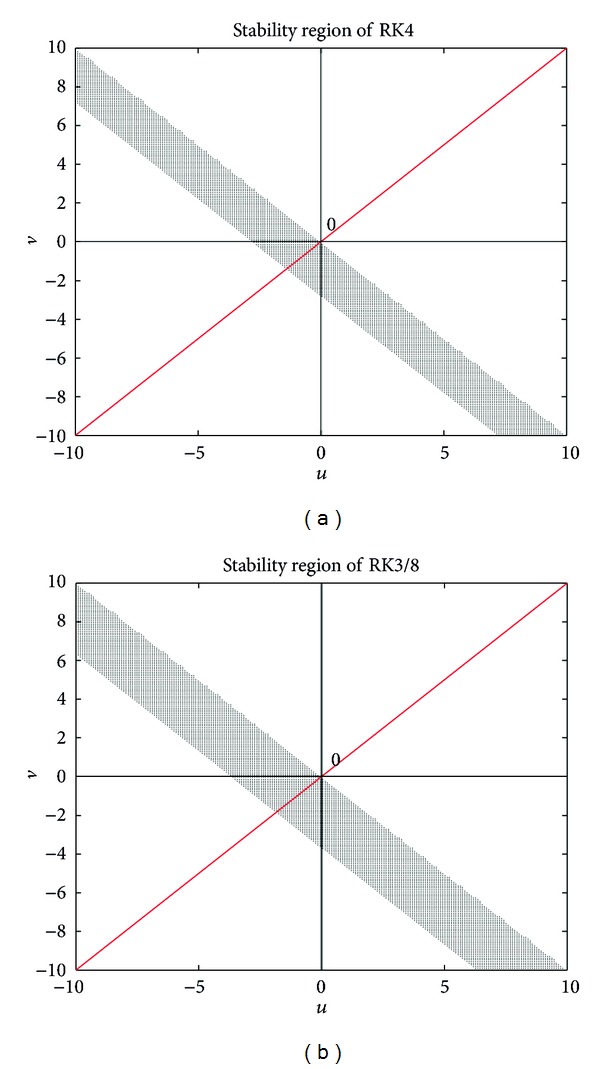
(a) Stability region of RK4 (left) and (b) stability region of RK3/8 (right).

**Figure 2:**
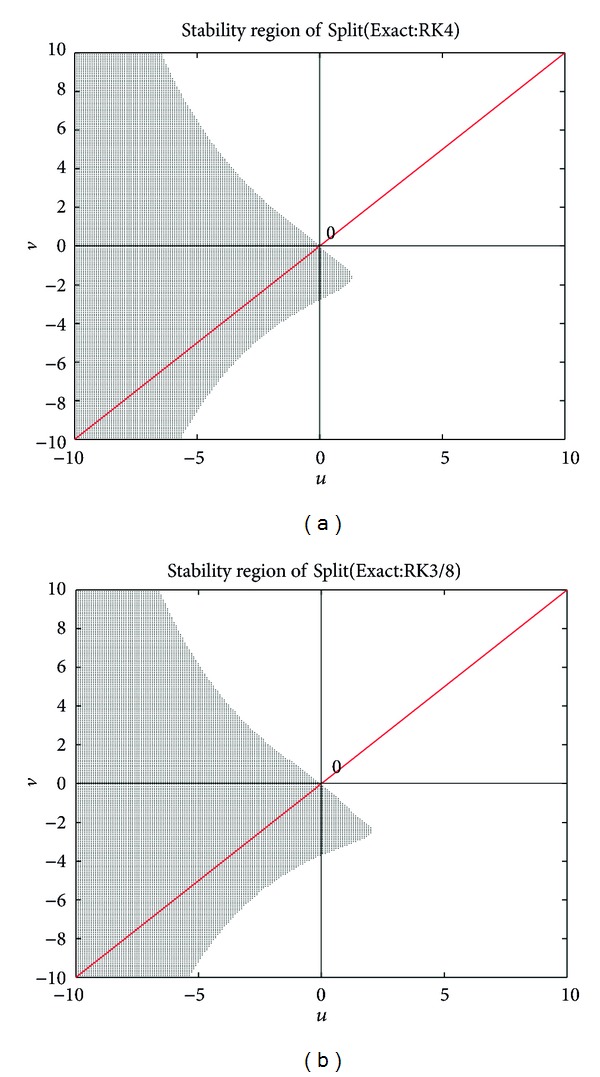
(a) Stability region of Split(Exact:RK4) (left) and (b) stability region of Split(Exact:RK3/8) (right).

**Table 1:** Parameter values for the one-gene network.

*α* = 3	*γ* = 1	*μ* = 1.5	*κ* = 5

**Table 2:** One-gene network: average errors for different stepsizes.

Stepsize	RK4	RK3/8	Split(Exact:RK4)	Split(Exact:RK3/8)
1.2	1.7733 × 10^0^	6.2404 × 10^−1^	1.3910 × 10^−3^	1.3910 × 10^−3^
1.5	3.2171 × 10^17^	1.2337 × 10^1^	1.1862 × 10^−3^	1.1862 × 10^−3^
2.0	3.3619 × 10^59^	6.0455 × 10^47^	5.4651 × 10^−4^	5.4651 × 10^−4^
10.0	8.7073 × 10^62^	7.6862 × 10^62^	1.1451 × 10^−7^	1.1451 × 10^−7^

**Table 3:** One-gene network: average errors for fixed stepsize *h* = 2 on different time intervals.

Time interval	RK4	RK3/8	Split(Exact:RK4)	Split(Exact:RK3/8)
[0,100]	3.3619 × 10^59^	6.0455 × 10^47^	5.4917 × 10^−4^	5.4917 × 10^−4^
[0,500]	4.5549 × 10^299^	2.0782 × 10^240^	1.1158 × 10^−4^	1.1158 × 10^−4^
[0,1000]	NaN	NaN	5.5903 × 10^−5^	5.5903 × 10^−5^
[0,1500]	NaN	NaN	3.7294 × 10^−5^	3.7294 × 10^−5^

**Table 4:** Parameter values for the two-gene network.

*λ*_1_ = 1.8	*γ*_1_ = 1	*κ*_1_ = 1	*δ*_1_ = 1	*θ*_1_ = 0.6542	*n*_1_ = 3
*λ*_2_ = 1.8	*γ*_2_ = 1	*κ*_2_ = 1	*δ*_2_ = 1	*θ*_1_ = 0.6542	*n*_2_ = 3

**Table 5:** Two-gene network: average errors for different stepsizes.

Stepsize	RK4	RK3/8	Split(Exact:RK4)	Split(Exact:RK3/8)
0.1	9.8188 × 10^−2^	9.3019 × 10^−2^	9.9338 × 10^−4^	9.9338 × 10^−4^
1.2	2.5157 × 10^−1^	5.5798 × 10^−2^	7.6803 × 10^−3^	7.6803 × 10^−3^
1.5	4.3953 × 10^0^	2.9958 × 10^0^	9.5832 × 10^−3^	9.5832 × 10^−3^
2.0	4.1821 × 10^24^	7.5238 × 10^17^	1.6686 × 10^−2^	1.6686 × 10^−2^
5.0	1.9692 × 10^69^	3.2225 × 10^68^	3.1227 × 10^−2^	3.1227 × 10^−2^

**Table 6:** Two-gene network: average errors for fixed stepsize *h* = 2 on different time intervals.

Time interval	RK4	RK3/8	Split(Exact:RK4)	Split(Exact:RK3/8)
[0,500]	4.4414 × 10^0^	1.5342 × 10^0^	1.9352 × 10^−3^	1.9352 × 10^−3^
[0,1000]	4.4396 × 10^0^	1.0172 × 10^0^	9.6936 × 10^−4^	9.6936 × 10^−4^
[0,1500]	NaN	2.3844 × 10^95^	1.1409 × 10^−3^	1.1409 × 10^−3^
[0,2000]	NaN	NaN	8.5613 × 10^−4^	8.5613 × 10^−4^
[0,2500]	NaN	NaN	6.8520 × 10^−4^	6.8520 × 10^−4^

**Table 7:** Parameter values for the p53-mdm2 pathway.

*s*_*m*0_ = 2 × 10^−3^ nM min^−1^	*k*_*a*_ = 20 min^−1^	*j*_*a*_ = 0.2 min^−1^	*γ* = 2.5
*s*_*m*1_ = 0.15 nM min^−1^	*k*_*c*_ = 4 min^−1^ nM^−1^	*j*_*c*_ = 2 × 10^−3^ min^−1^	
*s*_*m*2_ = 0.2 nM min^−1^	*k*_*u*_ = 0.4 min ^−1^	*d*_*m*_ = 0.4 min^−1^	
*s*_*p*_ = 1.4 nM min^−1^	*K*_*m*_ = 100 nM	*d*_*p*_ = 2 × 10^−4^ min^−1^	

**Table 8:** p53-mdm2 network: average errors for different stepsizes.

Stepsize	RK4	RK3/8	Split(Exact:RK4)	Split(Exact:RK3/8)
0.05	3.7686 × 10^−5^	3.7046 × 10^−5^	1.2091 × 10^−6^	1.2065 × 10^−6^
0.08	4.6118 × 10^0^	4.5057 × 10^0^	2.1383 × 10^−6^	2.1290 × 10^−6^
0.10	NaN	5.3550 × 10^0^	2.8098 × 10^−6^	2.7945 × 10^−6^
0.12	NaN	NaN	3.4986 × 10^−6^	3.4762 × 10^−6^
5.00	NaN	NaN	7.3001 × 10^−4^	3.8208 × 10^−4^

**Table 9:** p53-mdm2 network: average errors for fixed stepsize *h* = 10 on different time intervals.

Time interval	RK4	RK3/8	Split(Exact:RK4)	Split(Exact:RK3/8)
[0,100]	NaN	NaN	6.8810 × 10^−2^	6.6940 × 10^−2^
[0,500]	NaN	NaN	2.1634 × 10^−2^	2.0911 × 10^−2^
[0,1000]	NaN	NaN	1.1625 × 10^−2^	1.1214 × 10^−2^
[0,1500]	NaN	NaN	7.8314 × 10^−3^	7.5529 × 10^−3^
[0,2000]	NaN	NaN	5.8881 × 10^−3^	5.6786 × 10^−3^

## Appendix

### Stability Analysis of Runge-Kutta Methods and Splitting Methods

Stability analysis is a necessary step for a new numerical method before it is put into practice. Numerically unstable methods are completely useless. In this appendix, we examine the linear stability of the new splitting method constructed in Section 3. To this end, we consider the linear scalar *test equation*:

(A.1) $$\begin{array}{c} \dot{y}=\lambda y+\epsilon y, \end{array}$$

where *λ* is a test rate which is an estimate of the principal rate of a scalar problem, and *ϵ* is the error of the estimation. Applying the splitting method Φ_*h*_ (18) to the test equation (A.1), we obtain the difference equation

(A.2) $$\begin{array}{c} y_{n+1}=R\left(u,v\right)y_{n}, \end{array}$$

where *R*(*u*, *v*) is called the *stability function* with *u* = *λh* and *v* = *ϵh*.

Definition A.1 The region in the *u*-*v* plane

(A.3) $$\begin{array}{c} {ℛ}_{s}=\left\{\left(u,v\right)\;|\;\left|R\left(u,v\right)\right|\leq 1\right\} \end{array}$$

is called the *stability region* of the method and the curve defined by |*R*(*u*, *v*)| ≤ 1 is call the *boundary of stability region*.

By simple computation, we obtain the stability function of an RK method

(A.4) $$\begin{array}{c} R\left(u,v\right)=1+\left(u+v\right)b^{T}{\left(I-\left(u+v\right)A\right)}^{-1}e, \end{array}$$

where *e* = (1,1,…, 1)^*T*^, *I* is the *s* × *s* unit matrix and the stability function *R*(*u*, *v*) a splitting method Split(Exact:RK)

(A.5) $$\begin{array}{c} R\left(u,v\right)=\exp\left(v\right)\left(1+ub^{T}{\left(I-uA\right)}^{-1}e\right). \end{array}$$

The stability regions of RK4 and RK3/8 are presented in Figure 1 and those of Split(Exact:RK4) and Split(Exact:RK3/8) are presented in Figure 2. It is seen that Split(Exact:RK4) and Split(Exact:RK3/8) have much larger stability regions than RK4 and RK3/8. Moreover, the infinity area of the stability regions of Split(Exact:RK4) and Split(Exact:RK3/8) means that these two methods have no limitation to the stepsize *h* for the stability reason but for the consideration of accuracy, while RK4 and RK3/8 will completely lose effect when the stepsize becomes large to some extent. This has been verified in Section 4.
